# Recent Developments in Optical Detection Technologies in Lab-on-a-Chip Devices for Biosensing Applications

**DOI:** 10.3390/s140815458

**Published:** 2014-08-21

**Authors:** Nuno Miguel Matos Pires, Tao Dong, Ulrik Hanke, Nils Hoivik

**Affiliations:** IMST-Department of Micro- and Nanosystems Technology, Faculty of Technology and Maritime Sciences, Buskerud and Vestfold University College, Postboks 235, 3603 Kongsberg, Norway

**Keywords:** microfluidics, optical detection, electrochemistry, sensor integration, fluorescence, chemiluminescence, analyte detection, point of care

## Abstract

The field of microfluidics has yet to develop practical devices that provide real clinical value. One of the main reasons for this is the difficulty in realizing low-cost, sensitive, reproducible, and portable analyte detection microfluidic systems. Previous research has addressed two main approaches for the detection technologies in lab-on-a-chip devices: (a) study of the compatibility of conventional instrumentation with microfluidic structures, and (b) integration of innovative sensors contained within the microfluidic system. Despite the recent advances in electrochemical and mechanical based sensors, their drawbacks pose important challenges to their application in disposable microfluidic devices. Instead, optical detection remains an attractive solution for lab-on-a-chip devices, because of the ubiquity of the optical methods in the laboratory. Besides, robust and cost-effective devices for use in the field can be realized by integrating proper optical detection technologies on chips. This review examines the recent developments in detection technologies applied to microfluidic biosensors, especially addressing several optical methods, including fluorescence, chemiluminescence, absorbance and surface plasmon resonance.

## 1. Introduction

Detection of pathogenic organisms, hormones, or other medically relevant analytes still demands the development of innovative analytical devices with enhanced sensitivity, specificity, precision, speed and usability. Analysis of these analytes in the laboratory is still common practice. Although accurate and sensitive, laboratory methods usually require bulky and expensive instrumentation, labor-intensive sample preparation, with expert operators and personnel. Thus, the realization of miniaturized detection tools is a major driving force to achieve point-of-use, and real-time monitoring of real samples. Device miniaturization can be achieved using Lab-on-a Chip (LOC) Technology which integrates several laboratory functions on a single chip. This technology employs microfluidics and deals with the handling of small volumes of fluids in microchannels. A variety of academic proof-of-concept studies have shown the advantages of LOC systems over laboratory tests [1–5]. These advantages include reduced sample and reagent consumption, automation, and fast detection times.

Coupling a detector to a LOC is critical for any analytical device. Most applications in clinical diagnostics and environmental monitoring demand robust, cost-effective detection devices for the rapid and sensitive analysis of analytes. For instance, effective surveillance for waterborne pathogens could be achieved by developing low-cost, portable sensors for the real-time detection of only 10–100 organisms in a sample [6–8].

A number of detection technologies have been demonstrated in LOC devices, including electrochemical [9], mechanical [10] and optical methods [11]. Previously, Schwarz and Hauser [12] have addressed the value of electrochemical and optical architectures for designing sensitive microfluidic analytical systems. These detection architectures were further reviewed by Mogensen *et al.* [13]. Later, Waggoner and Craighead [14] reviewed innovations on micro- and nanomechanical sensors for general chemical and biological analysis. A new study addressing all electrochemical, mechanical and optical detection methods would be far more attractive to clinical researchers, environment authorities or companies active in biotechnology. Here, a review of popular microfluidic detection technologies reported in the past five years is presented.

## 2. Overview of Detection Methods in Microfluidic Devices

The small sample volumes often encountered in microfluidic devices pose an important challenge to the detector. The detection mechanism should be highly sensitive and specific to the target analyte. An ideal detector would require rapid response, minimal sample preparation and low-cost fabrication. Moreover, low power consumption, compactness, automation, and potential for realizing multiplex analysis would be desired characteristics for point-of-use applications [15,16]. The electrochemical, mechanical and optical detection for LOCs are summarized and commented in Table 1.

### 2.1. Electrochemical

Electrochemical detection involves interaction of chemical species with electrodes or probes. This interaction results in variations of electrical signals, such as potential or current, which enables quantitative analysis of target analytes. The electrochemical phenomenon deals with two major effects: (i) chemical reactions are promoted by passing an electrical current through the electrode system; or (ii) electrode responses are triggered due to specific chemical reactions. These effects usually occur in an electrolytic cell. Reactions of oxidation and reduction occurring at the surface of the electrodes are the basis for electron transfers between the electrolyte (sample) and the electrodes. In a typical electrolytic cell, the electrode system is formed by the working electrode where detection of a certain analyte is analyzed, and the reference electrode where a standard oxidation/reduction is conducted [17]. The setup is completed by adding a third electrode, entitled counter electrode. This counter electrode is used to minimize the electrical current flowing through the reference electrode, thus maintaining its potential constant during the operation of the electrolytic cell.

Although traditional, the three-electrode setup is still used in modern electrochemical biosensors. Srivastava *et al.* [21] have developed an electrochemical microfluidic biosensor comprising of reference (Ag/AgCl), counter (ITO) and working (TiO_2_-ZrO_2_/ITO) microelectrodes. This sensor employed the amperometric detection principle for quantification of urea. Detection was conducted by measuring the increase in the peak current magnitude of cyclic voltammetric responses due to increased urea concentrations. The three microelectrodes were prepared on a glass substrate and further integrated with low-cost polydimethylsiloxane (PDMS) microchannels. The microfluidic sensor showed highly selective and linear with a detection sensitivity of 2.74 mA [log·mM]^−1^·cm^−2^ and detection limit of 0.44 mM. Response times of around 10 s were achieved for experiments conducted in phosphate buffer saline (PBS) containing 0.9% NaCl at pH 7.0 strictly controlled. The shelf-life of the sensor was about four weeks.

MicruX [22] now commercializes electrochemical flow-cells with similar microelectrode configuration. These cells incorporate standard microfluidics, and amperometric measurements are performed requiring low sample volumes (<20 μL). Ideal in-the-field testing devices should perform analysis with minimized waste of sample and reagent volumes. This commercial microfluidic platform is described to have a dead-volume of <500 nL. The microfluidic channels are arranged in a methacrylate substrate where the fluid flow is directed to be perpendicular to the surface of the working electrode. This approach is claimed to maximize mass transfer between the sample and electrode surface, and consequently improve the sensitivity of electrochemical detection. The fluidic substrate is reusable while the electrodes can be easily replaced. The possibility of electrode replacement may compensate the short shelf life of the sensor. Nevertheless, the platform is fully compatible to any potentiostat (control circuitry) because of the integrated universal USB connector.

Modification of the electrode configuration is one way to improve the sensitivity of amperometric detection. Wongkaew *et al.* [18] have proposed a novel electrochemical microfluidic biosensor employing interdigitated microelectrode arrays. This microelectrode geometry corresponds to a pair of microband array electrodes that intersect with each other. Adjacent electrode fingers form micro-sized gaps which allow an increase of the diffusion flux of chemical species, thus leading to an enhanced collection efficiency and higher signal amplification. The electrodes made of 200-nm thick gold were deposited onto thiol-functionalized poly(methyl methacrylate) (PMMA) pieces following e-beam evaporation and wet-etching processes. Hot embossing, a superior tool for mass production of plastic microchips [23], was used for microchannel fabrication in PMMA. The device was applied to the quantification of single-stranded DNA sequences from waterborne *Cryptosporidium parvum*. Employing probe-coated paramagnetic beads [24] and probe-tagged liposomes entrapping ferri/ferro hexacyanide, DNA was detected within 250 s and with a limit of detection of 12.5 μM. Furthermore, the device was comprised of multiple channels within the same chip, which may lead to an increase of the test throughput.

Further studies have demonstrated the incorporation of electrochemical detection in paper-based microfluidic devices. This approach is specifically targeted to develop to easy-to-use, very low-cost, and fully portable solutions for point-of-care testing [25]. Microfluidic channels are arranged on cellulose fiber-based paper using photolithography, while electrodes are fabricated on paper employing screen-printing technology. Enzymes and DNA strands can be immobilized onto the surface of the screen-printed electrodes, and the immobilized DNA can serve as capture probe for the target analytes. Determination of glucose, lactate, and uric acid in biological samples was demonstrated with paper-based microfluidic devices [26]. Moreover, analyte detection was shown in range of few mM in human serum samples.

Electrochemical detection is an attractive option to miniaturized analytical systems. High sensitivity, good precision, cost effectiveness, easy incorporation into microfluidic chips, and low power consumption are key advantages of electrochemical microfluidic systems [27,28]. However, although the electrochemical response is independent from the optical path length which is encountered in optical based techniques, the detection electrodes are highly influenced by variations of temperature, pH and ionic concentrations which limit the shelf life of the devices.

### 2.2. Mechanical

Micro- and nanometer scale mechanical systems, mainly cantilevers, have been studied for decades for sensor applications. Cantilever technology has shown its value in accurate sensing of biomolecules [14]. Cantilever-based devices generally operate in two different modes upon analyte binding: (i) static deflection, where binding on one side of a cantilever causes unbalanced surface stress resulting in a measurable deflection; (ii) dynamic, resonant mode, where binding on a cantilever causes variations of its mass and consequently shifts the resonant frequency. Various physical methods can be employed for actuating or sensing cantilever motion, including mechanical, optical, electrostatic, and electromagnetic methods. Cantilever-based devices can be realized with different shapes and sizes using conventional MEMS photolithography processes, and bulk or surface micromachining. The flexibility of device design indicates the possibility of incorporation in microfluidic systems and miniaturized LOCs. With appropriate chemical functionalization the devices can specifically detect different chemical and biological entities. Mechanical-based detection may require no labelling of biomolecules. Often, labels make the detection method more complicated, time-consuming and costly, and could interfere with the function of antigens or antibodies. Other characteristic of cantilever technology is the potential to fabricate large arrays of sensors for multi-molecular sensing [19].

Deflection cantilever detection involves generation of surface stress which results in increased deflection at the free end of a flexible cantilever beam. Therefore, if an analyte binding to a functionalized cantilever surface induces a surface stress, cantilever beams will bend up or down, which can be recorded. The beam deflection is commonly measured using optical reflection; a laser is focused on the cantilever and reflected onto a position sensitive detector. Varying detector signals will indicate different beam curvature radii. Deflection-based mechanical sensors have been exploited for the detection of biomarkers, toxins and pathogenic organisms. Van den Hurk *et al.* [29] have used silicon cantilever arrays to detect γ-interferon, an important biomarker protein for monitoring multiple sclerosis. The surface of the silicon cantilevers were functionalized with antibody using glutaraldehyde, Prolinker B and 1-ethyl-3-[3-dimethylaminopropyl]carbodiimide hydrochloride (EDC)/N-hydroxysulfosuccinimide (Sulfo-NHS) linking procedures. One-sided functionalization of the cantilever may be achieved by gold coating the silicon beam surface [30]. Despite the strong affinity of gold surfaces to bind biomolecule probes, these layer structures can be significantly affected by temperature fluctuations or other environmental conditions, resulting in background noise. Immobilization of DNA probes onto a silicon cantilever was recently demonstrated [31]. This deflection-based cantilever sensor showed a resolution of 0.2 nM for the determination of oxytetracycline. Furthermore, Anderson *et al.* [32] have shown the integration of a mechanical sensor consisting of silicon microcantilevers with standard PDMS microfluidic channels. However, the microfluidic integration of a highly sensitive deflection-based sensor requires special attention. The accuracy of a deflecting cantilever is proportional to the square of the beam length. Longer cantilevers pose challenges to micromachining techniques, as the risk of stiction failure increases. In addition, the application of these cantilevers to biomolecule detection in fluids would require external fluid cells for precise control of flow rate and additional encapsulation techniques. These challenges would make the micromechanical devices with integrated microfluidics more complex and difficult to use in low-cost, disposable microfluidic sensors.

Resonant micro- and nanomechanical sensors offer other way to achieve highly sensitive label-free mass detection. These sensors are generally composed of cantilevers operated in the dynamic mode and are excited at a stable resonant frequency. As a deflection-based sensor, a resonator can be functionalized to specifically bind a particular analyte. The binding events cause changes in the resonant frequency of the devices, which are proportional to the amount of bound analyte. Using frequency shift measurements, detection limits in the attogram range [33] can be achieved with nanocantilevers when operated in vacuum. Another unique characteristic of resonant cantilevers is the availability of various techniques used to actuate and detect resonance. The use of piezoelectric materials is popular for both resonance excitation and detection. Sharma *et al.* [34] have developed self-exciting and self-sensing piezoelectric cantilevers consisting of lead zirconate titanate (PZT). This cantilever system was coupled to a flow cell, and chemical detection was demonstrated in ethanol solution. Further, gold was sputtered onto the tip of the PZT cantilever to detect DNA hybridization [33]. A decrease in the resonant frequency of the PZT was related to an increase of cantilever mass due to the interaction between biochemical targets and DNA probes immobilized onto gold-modified PZT surface. Normally, a sinusoidal electric field is applied to cause cantilever deformation at a frequency equal to the excitation frequency. In addition to piezoelectric actuation, Ricciardi *et al.* [35] used an optical readout composed of a laser diode and position sensitive detectors for monitoring cantilever resonance response. The cantilever devices were fabricated from silicon-on-insulator wafers using wet etching, photolithography and reactive ion etching. Standard PDMS microfluidic channels were employed for the integration with the silicon cantilevers. In-liquid analysis of biomarkers was conducted on top of the cantilevers through modification of the cantilever surface with protein G and biomarker-specific antibody. The use of both optical excitation and detection would greatly simplify the fabrication and handling of resonators [14]. A focused laser beam can act as a localized heat source which thermally excites oscillation. This oscillation was measured at the free end of a silicon resonant cantilever by laser Doppler velocimetry [36]. Another advantage to optical excitation is its application to a wide variety of device geometries, leading to innovative resonant sensors unhindered by electrical integration requirements. The aforementioned examples have shown the integration of resonant mechanical sensors to LOCs; however, the detection sensitivities are limited by mechanical losses associated with viscous damping.

### 2.3. Optical

Because of the limitations of both electrochemical and mechanical techniques, optical detection is preferred for robust, sensitive LOCs. Furthermore, optical detection has been the most widely used technique for quantitative proteomic analysis [37] and infectious disease diagnostics [38], due to the ubiquity of optical instrumentation in the laboratory. Conventional optical detection methods, including absorbance, fluorescence, chemiluminescence, and surface plasmon resonance (SPR), have all been applied in microfluidic biosensors. While microscopes, lasers, spectrophotometers, charge-coupled devices (CCDs) and photomultiplier tubes (PMTs) can be precisely coupled to LOCs [12,39–43], these systems are difficult to miniaturize into low-cost, portable detection devices. Alternatively, optoelectronic technology (waveguides, photodiodes) has been successfully integrated in microfluidic systems [44–48] in order to reduce the cost of diagnostic platforms. Integrated optical microfluidic platforms would also offer potential for achieving ultra-sensitive detection of bio-analytes at the micro and nano device scales. The next section summarizes recent developments in optical detection technologies for LOCs, and a list of these techniques is shown in Table 2.

## 3. Optical Microfluidic Detection Techniques

### 3.1. Fluorescence

Fluorescence is the result of a three-stage process (excitation, excited-state lifetime, fluorescence emission) that occurs in certain molecules called fluorophores or fluorescent dyes. A fluorescent dye is a small molecule, protein, or quantum dot which can label proteins, nucleic acids, or lipids. The availability of highly sensitive, highly selective fluorescent labelling techniques makes fluorescence a widely used optical method for molecular sensing in microfluidic systems. Fluorescence detection often involves: (1) an excitation light source; (2) a fluorophore; (3) wavelength filters to isolate emission photons from excitation photons; (4) a detector that registers emission photons and produces a recordable output, generally an electrical signal.

Although microscope optics, CCDs, or PMTs commonly add substantial size and complexity to the detection systems, coupling these off-chip approaches to microfluidic chips still continue. Lee *et al.* [41] have developed an optofluidic microscope technique for imaging *Giardia lamblia* cysts, a disease-causing protozoan species that is frequently found in environmental waters. Imaging is conducted by scanning the target analytes across a slanted array of holes, and images of the target are obtained by measuring the time-varying light transmission changes through the holes. A resolution of 0.8 μm was achieved by use of high-density image array sensors made in complementary metal-oxide-semiconductor (CMOS) technology. A conventional optical microscope was used to illuminate the CMOS image sensors which were attached to standard PDMS microfluidic channels. Fluorescent imaging is less likely to detect bacteria or viruses. Ramalingam *et al.* [49] presented a real-time pathogen detection instrument incorporating polymerase chain reaction (PCR) assays in PDMS-glass microfluidic chips. Parallel detection of genomic DNA from *Aeromonas hydrophilia*, *Klebsiella pneumonia*, *Staphylococcus aureus* and *Pseudomonas aeruginosa* was demonstrated using a CCD camera. The optical detection system was specifically designed to measure the fluorescence of EvaGreen, a DNA intercalating dye. Although the reported instrument uses no external pumps and valves, the microfluidic PCR assay need a localized thermal cycling scheme which limits their application to point-of-care analysis. Thermal cycling would require additional operation steps and make the microfabrication of the devices more complex. Nucleic acid sequence-based amplification (NASBA) may be an alternative to PCR for LOCs. NASBA involves isothermal amplification of mRNA sequences for DNA detection. It can be realized on-chip using conventional fluorescence scanners such as microarray readers [64,65]. Simultaneous detection of waterborne *E. coli* and rotavirus was conducted in disposable PMMA microfluidic chips by coupling the NASBA scheme to highly sensitive immunological assays [64].

**Figure 1.**
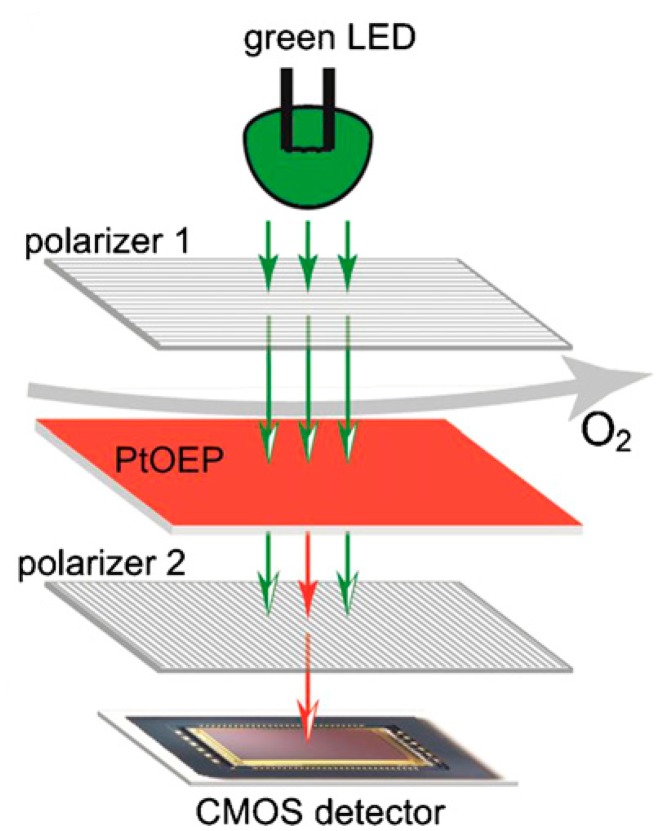
Conceptual design of a fluorescence based detection device showing a light source (LED), photodetector (CMOS), polarizers and O_2_ sensitive PtOEP film arranged in a portable O_2_ sensing system. Reprinted from [66], with permission from Elsevier.

Off-chip readout methods often require long working distances and include external lenses, thus leading to high optical losses and decreased signal-to-noise ratios [57]. Moreover, the detection systems are generally large, which heavily limit the portability of the microfluidic devices. Compact systems would be realized by incorporating lasers, filters, fluid channels and detectors into a single microchip. Shen *et al.* [66] presented a portable optical oxygen sensor integrating a CMOS detector and polarizer filters. The arrangement of the optical components for fluorescence detection is shown in Figure 1. The sensor exhibited sensitivities comparable to that of macroscale benchtop sensor systems [66]. Other portable biosensing platforms were proposed for on-site monitoring of hormonal compounds in environmental waters [50]. The platform integrated diode lasers and fiber probes in a glass flow cell. The excitation light from the lasers was coupled to fiber probes. The incident light propagated along the length of the probe via total internal reflection. The evanescent wave generated at the surface of the probe then interacted with the surface-bound fluorescently labeled analyte complexes and caused excitation of the fluorophores. The collected fluorescence was filtered by means of a bandpass filter and detected by conventional inorganic photodiodes. A detection limit of 2.1 nM was achieved with the portable platform employing aptamer-based assays. On-chip CMOS and silicon photodetectors are capable of providing high detection sensitivity for low analyte concentration; however, these detectors are too expensive and complicated to fabricate as an integral part of a disposable sensor. Organic photodiodes (OPDs), in comparison, may offer the best potential for future LOCs, as they can be realized onto glass or plastic chip substrates using simple low-cost fabrication methods, including spin-coating, inkjet printing, and spray-coating. Pais *et al.* [67] have reported a disposable LOC with integrated OPDs for the first time. A CuPC/C_60_ thin-film OPD was used as a photodetector for fluorescence detection of Rhodamine 6G and fluorescein, while the excitation source was a thin-film organic light-emitting diode (OLED) made of NPB/Alq3. The on-chip approach showed detection limits as low as 100 nM. Other compact and inexpensive LOC for on-chip fluorescence analysis was developed employing the CuPC/C_60_ thin-film OPD [68]. This microfluidic device detected resorufin with a resolution of 5.0 μM.

The sensitivity of fluorescence detection in microfluidic systems is sometimes compromised by background signals, which may originate from autofluorescence of sample constituents. Furthermore, the fluorescent dyes are costly, have a limited shelf life, and are often influenced by pH. The labelling procedure also involves complex fluid handling, thus hindering automation of a rapid assay.

### 3.2. Chemiluminescence

**Figure 2.**
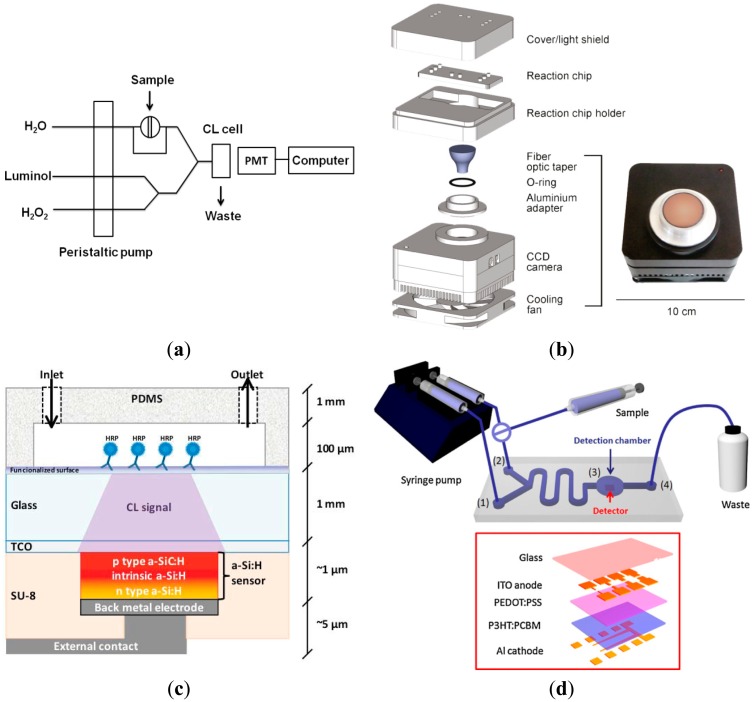
Methods of chemiluminescence (CL) detection in microfluidic systems. (**a**) Flow injection system for CL analysis using PMT technology [53]; (**b**) Microfluidics-based device incorporating a thermoelectrically cooled CCD camera. Reprinted with permission from (with permission from [73]); (**c**) Integrated opto-microfluidic sensor with a hydrogenated amorphous silicon (a-Si:H) photodetector prepared onto a glass substrate covered by a transparent conductive oxide (TCO) film (with permission from [55]); (**d**) Integration of an organic P3HT:PCBM photodetector to a CL reaction chamber. Two inlets (1 and 2) and one outlet (4) were arranged in a microfluidic channel containing the detection zone (3) (Reprinted from with permission from [78]).

Chemiluminescence is also an attractive optical method for analyte detection in which target binding cause photochemical emission, either directly or with the help of an enzyme label. The advantage of this technique for LOCs is that excitation light sources and emission filters are not required, thus minimizing likely background interferences. However, highly sensitive detectors are typically demanded. In the laboratory, computerized ultra-weak luminescence analyzers are often employed to measure the emitted photons generated during the chemiluminescent reactions [69–71]. Yu *et al.* [70] developed a disposable microfluidic paper-based analytical device for uric acid determination. The chemiluminescence of rhodanine and H_2_O_2_ was monitored using a portable luminescent analyzer, and a detection reproducibility of over 10 weeks was obtained. The shelf life of this chemiluminescent paper-based device would be much larger than that of an electrochemical-based paper device. Using a similar luminescent analyzer, Ambrosi *et al.* [69] have exploited the chemiluminescence of horseradish peroxidase (HRP) and 3,3′,5,5′-tetramethylbenzidine (TMB) enzyme substrate to detect a breast cancer biomarker in real blood samples. The method showed a superior detection sensitivity comparing to a classical enzyme-linked immunosorbent assay (ELISA) test. Furthermore, the assay time of this method was only 5 min, well below to that of the classical ELISA. Enhanced chemiluminescent ELISA sensitivities could also be achieved by coupling external CCD or PMT sensors to microfluidic channels. The carcinoembryonic antigen was targeted in a sandwich chemiluminescent immunoassay, and detected by an external PMT (see Figure 2a) at a resolution of 20 pg/mL [53]. Furthermore, sandwich chemiluminescent immunoassays were exploited to detect Staphylococcal Enterotoxin B in disposable polycarbonate plates [72]. Employing gold nanoparticles and an external CCD camera, a detection limit of 10 pg/mL for this foodborne toxin, which was around 10 times more sensitive than traditional ELISA, was achieved. Chemiluminescence detection may ensure the portability of CCD-based microfluidic sensors. Using CCD technology, Roda *et al.* [73] have proposed a relatively large-sized portable device for chemiluminescence imaging (Figure 2b) of a wide range of proteins and nucleic acids. Further miniaturization would lead to faster analysis times and more autonomous analytical devices.

Miniaturized detection systems can be realized by integrating silicon photodiode arrays, ring resonators [74,75], or photonic crystals [76] into LOCs. Using silicon as the material platform offers high sensitivity and potential for concurrent detection of multiple analytes via standard CMOS technology. Arrays of hydrogenated amorphous silicon (a-Si:H) photodiodes have been popularized as detectors for chemiluminescence detection [55,77]. A schematic overview of the microfluidic integration of a-Si:H sensors is shown in Figure 2c. This scheme was reported to achieve practical detection limits in the attomole range. The a-Si:H photodiodes are commonly fabricated by RF plasma enhanced chemical vapor deposition and post-processed by a number of photolithography and reactive ion etching processes. The several processing steps prevent the realization of a-Si:H photodetectors in disposable, point-of-use microfluidic devices. OPDs often involve a two-step fabrication process, including spin-coating of polymer films and thermal evaporation of thin electrodes. The low-cost OPDs have already been exploited in miniaturized chemiluminescence sensors envisaging the realization of simple, inexpensive devices for use in the field. Figure 2d shows a schematic of the P3HT: PCBM OPD reported for microfluidic detection of antioxidants [78].

Sensitive antioxidant assays were developed in standard PDMS microfluidic channels, exploiting the chemiluminescence reactions of peroxyoxalate with hydrogen peroxide in the presence of 9,10-diphenylanthracene. Wojciechowski *et al.* [57] has further integrated the P3HT:PCBM OPD to a real hand-held reader. The OPD and a PDMS reservoir formed the disposable part of the portable analytical device, in which pathogen detection immunoassays were conducted using the chemiluminescent system of HRP and luminol/peroxide/enhancer cocktail. Staphylococcal Enterotoxin B was detected at concentration as low as 500 pg/mL [57], which is still inferior to the aforementioned CCD-based sensors. Besides the use of the CuPC/C_60_ OPD in fluorescence detection, this OPD was successfully employed in chemiluminescent flow-through immunossays. IgA, a marker of human stress, was detected at a limit of 16 ng/mL, using the luminescent enzymatic reaction of HRP and Amplex Red. The photosensitivity of the CuPC/C_60_ OPD remained relatively constant for around one year [68]. The performance of polymer photodetectors is still inferior to that of their silicon counterparts. The realization of detectors with comparable performance to the silicon photodiode at price of a simple photoresistor would be a tremendous advance to optical LOCs [79]. Derivatives of poly(2,7-carbazole) and poly(4,8-bis-alkyloxybenzodithiophene) may form OPDs with enhanced response stability [80,81] and higher light absorption compared to P3HT-based sensors [82,83].

Chemiluminescence offers a simple detection method for general LOC applications. The detection requires no complex instrumentation which greatly decreases the cost of the systems and enhances the portability of the analytical devices. However, the development of low-cost sensitive photodetectors is still necessary to the successful adoption of chemiluminescence microfluidic sensors in applications requiring disposable and easy-to-use devices.

### 3.3. Absorbance

Absorbance detection methods involve test of the analyte concentration by measuring the absorbance/attenuation of a specific wavelength of incident light. In the laboratory the light attenuation is commonly measured using UV absorption spectroscopy. UV absorption was exploited on-chip by Gustaffson *et al.* [84]. UV-transparent SiO_2_ waveguides were arranged in a silicon substrate and integrated to microfluidic channels. This work was to develop an on-chip approach of electrochromatographic separation. Miniaturization provides a number of benefits for UV absorption based techniques including enhanced efficiencies, reduced analysis times, and reduced power consumptions.

Other miniaturized absorbance detection system was developed by integrating a CMOS image sensor with embedded RGB Bayer filters to typical PDMS microfluidic channel bonded to a glass coverslip [58]. Solutions of eosin Y, a red dye with its maximum absorption in aqueous solution at ∼515 nm, were firstly evaluated. Further, the authors have performed colorimetric glucose assays in which the conversion of glucose into gluconic acid was proportional to the oxidation of o–dianisidine to form a colored product, the absorbance of which was measured with the integrated CMOS absorbance detector. Detection results were similar to that obtained with a conventional spectrophotometer. In several application cases, visually observable changes in optical density or colour are sufficient for diagnosis. Lei *et al.* [85] developed a colorimetric immunoassay for human IgG detection employing gold nanoparticles that signalized antigen-antibody binding events. The results of immunoassay were represented by the level of color intensity, which was easily observed by a regular camera or naked eye. Qiuhua *et al.* [86] have also used gold nanoparticles in molecular assays for colorimetric detection of Hepatitis E virus (HEV) RNA. Visual detection of 101 RNA copies was achieved.

A major drawback of absorbance-based detection in microfluidics is that as sample volumes decrease, the optical path length through the sample decreases, and this directly impacts sensitivity as described by the Beer-Lambert law. Despite the relatively poor sensitivity of microfluidic absorbance detection compared to fluorescence, its instrumentation simplicity gives it an advantage in applications requiring point-of-use analysis. Indeed, a number of absorbance-based microfluidic point-of-care products are available. The Claros test by Opko Health, for example, performs multiplex and quantitative immunoassays based on absorbance measurements for monitoring disease markers [87]. The test uses a silver enhancement chemistry to amplify the immunoassay signal output which can be measured by variation in optical density [88]. Multiple immunoassay protocols typically encountered in the ELISA test are replicated within the Claros cartridge device. Rapid absorbance-based detection of multiple cardiac markers, such as Myoglobin and Troponin I, can be performed by Cardiac Reader^®^ [89] and Cardiac STATus^®^ [90] from Roche and Nexus Dx, respectively.

### 3.4. SPR

**Figure 3.**
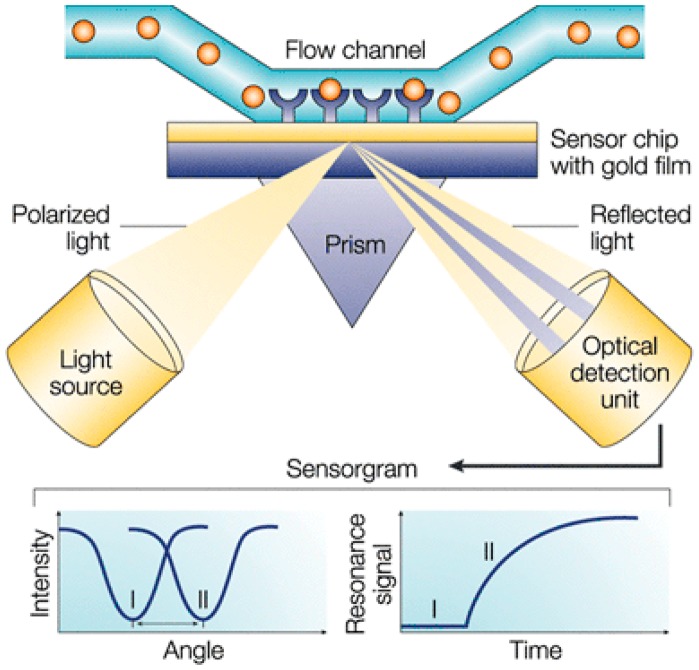
Setup of a microfluidic SPR biosensor. Reprinted by permission from Nature Publishing Group: [91], copyright (2002). The configuration encompasses a light source, a prism and a detector, all coupled to a metal-coated sensor microfluidic chip. SPR detection involves variation in the refractive index in the immediate vicinity of the metal layer of the sensor chip.

The principle of SPR biosensing relies on the detection of a refractive index change at a metal surface (typically gold) which is functionalized with probes molecules (e.g., antibodies). When light is incident on a thin metal film at a specific angle through a prism, it excites a propagating surface plasmon at the surface of the metal. At this angle, the reflectance intensity decreases sharply, and SPR will be observed as a shadow in an image detector. This SPR angle is highly dependent on the mass of material on the opposite side of the metal surface. The angle shifts (from I to II in the lower left-hand diagram of Figure 3) when the target analytes bind to the probe-functionalized metal surface and change the mass of the surface layer. This mass change is thus transduced in change of resonant angle which can be monitored in real time as a plot of resonance signal (proportional to mass change) *versus* time (see Figure 3).

SPR is a ubiquitous label-free detection technique in the laboratory. Several laboratory-scale SPR instruments for immune-sensing and DNA hybridization detection are currently used, most notably the Biacore from GE Healthcare [92,93]. Recently, efforts have focused on reducing the size and complexity of SPR sensors by integrating microfluidics. Frasconi *et al.* [94] demonstrated the use of an Eco Chemie Autolab SPR system for the immunological detection of cortisol and cortisone in saliva and urine. Using polycarboxylate-hydrogel-based coatings for antibody immobilization onto gold disks, the authors showed a detection limit of less than 10 μg/L, sufficiently sensitive for both clinical and forensic use. Besides, the SPR sensor was stable and responsive for as many as 100 determination cycles. The SPRi-Lab+ instrument from GenOptics, equipped with an LED source, a CCD camera, and a microfluidic cell, was used by Foudeh *et al.* [63] for the molecular detection of RNA sequences from waterborne *Legionella pneumophila*. This pathogenic organism is the causative agent of Legionellosis, responsible for fatality rates over 10% within hospital and industrial outbreak settings. The reported SPRi instrument was ultra-sensitive to RNA of *L. pneumophila* (0.45 fM) employing molecular hybridization and further SPR signal amplification with streptavidin-coated quantum dots.

Krupin *et al.* [61] have recently developed a miniaturized SPR platform, incorporating surface plasmon waveguides, for biosensing of cells and proteins. The biosensor consisted of 5-μm wide, 22-nm thick Au stripes embedded in polymer (CYTOP^TM^) with microfluidic channels etched into the top cladding. This device performed selective capture of cells in buffer by the functionalization of the Au waveguides with antibodies against red blood cells. Furthermore, bovine serum albumin was targeted on a carboxyl-terminated self-assembled monolayer prepared on the waveguides and detected with a resolution of ∼12 pg/mm^2^. Another miniaturized platform was developed by Escobedo *et al.* [95]. A unique microfluidic concentration gradient generator, made of PDMS, incorporated a nanohole array SPR sensor, fabricated by focused-ion beam milling on commercial Au-coated glass. Escobedo *et al.* have stated that the SPR measurements with the nanohole structures can be accomplished using simpler optical arrangement, and cheaper light sources and detectors. One ovarian cancer marker (r-PAX8) was detected at a limit of 5 nM; however, no test with clinical samples was reported.

Moreover, parallel detection of multiple analytes via SPR was demonstrated using an integrated microfluidic array [62]. According to the reported design, high-throughput SPR measurements can be conducted on 264 element-addressable chambers, fabricated in PDMS using soft lithography techniques. In theory, the array is capable of analyzing up to 264 different protein targets in a single experiment. The complex SPR chip also contained one micropump and thousands of microvalves that were used to isolate and control the flow of fluids precisely. Tests with human α-thrombin immobilized on the sensor surface revealed detection sensitivities down to ∼nM range in immunoassays performed in a 700 pL chamber.

The sensitivity of SPR detection can be enhanced by use of gold nanoparticles in the assays performed on the sensor surface. In the work developed by Uludag *et al.* [96], the surface of gold nanoparticles was modified with antibody against prostate-specific antigen. Detection of the target analyte was conducted by forming an immunological “sandwich” between the antibody-modified nanoparticles and the capture antibodies previously immobilized on the surface of the SPR sensor. The limit of detection of this SPR immunoassay was in the range of ∼pM [96]. However, there was a tradeoff between nanoparticle diameter and sensitivity. With 40 nm diameter nanoparticles, the detection limit was about 10 times smaller than that for Au particles with 20 nm diameter. The assay sensitivity obtained in 75% human serum was well below the threshold value for prostate cancer detection.

Other SPR concepts were proposed for reducing the complexity of the SPR instrumentation. Sensors based on the localized surface plasmon resonance (LSPR) phenomenon exploit the collective resonant oscillation of conduction electrons at the surface of a metal nanoparticle under the perturbation of incident light. Whereas conventional SPR sensing requires a prism or grating coupler to excite propagating plasmons on the metal surface, LSPR sensing requires no special coupling instrumentation and is typically performed with a white light source. Furthermore, the sensitivity of LSPR sensors is less likely to be affected by background interferences, for instance from non-specific binding of non-targeted molecules, because of its near-field phenomenon (<20 nm) [97]. Label-free biosensors exploiting LSPR has been demonstrated for detecting antigen/antibody binding, DNA hybridization, and small molecules. Examples of LSPR biosensing can be seen in the works of Huang *et al.* [98] and Piliarik *et al.* [99].

Although SPR often shows high sensitivity to multiple analytes, the instrumentation required for SPR measurements is complex, and the fabrication of microfluidic devices with integrated SPR sensors is still very expensive. In addition, the strong influence of temperature to the detection performance and the required use of gold surfaces or nanoparticles prevent the realization of SPR biosensing in disposable, point-of-care devices. The technique is most likely to be conducted in the laboratory due to the difficulty of realizing robust, cost-effective sensors with integrated microfluidics.

## 4. Conclusions

Microfluidics is, in its essence, a technology with a primary goal of providing miniaturization and automation of typical laboratory methods. To realize microfluidic diagnostic devices, there have been significant advances in microfluidic detection technologies; however, there are still challenges related to developing fully integrated functioning detection devices that provide real clinical application value. The main challenges towards achieving this practical goal are listed as following: (1) Development of practical LOC components and assay procedures. These areas include new methods for sample collection, reagent storage, analyte targeting, signal amplification, and working with complex sample specimens (including blood, urine, and saliva). Such components and procedures are necessary for developing a robust, integrated microfluidic device with clinical relevance; (2) Realization of robust system integration. For the last five years, efforts have focused on developing innovative sensing strategies for integrated LOCs. However, the application of traditional laboratory techniques as off-chips approaches to LOCs still continues. The robustness of the microfluidic detection systems would require the compatibility of bench-top methods to standard microfluidic structures, leading to autonomous miniaturized devices; (3) Lack of sufficient testing with complex sample specimens, once an integrated device is built. Validation of the on-chip detection approaches against real samples is a requirement for successful adoption of these systems by the clinical personnel.

A variety of techniques have been proposed for analyte detection in LOCs, including electrochemical, mechanical, and optical methods. The proper integration of these techniques in microfluidic chips would address most of drawbacks seen when performed laboratory analysis. This would bring the end goal of developing practical microfluidic devices into reality by performing sample-to-result diagnostic tests with low detection limits in a short time. Despite the recent advances on sensitive electrochemical and mechanical methods, those still present important drawbacks for realizing disposable microfluidic devices. Electrochemical detection often involves biosensors with low shelf life, while mechanical resonance detection requires devices realized by expensive micro/nanofabrication processes. Optical detection remains an attractive technique for microfluidic analysis of pathogens and proteins, although integrating sensitive optical detectors in inexpensive microfluidics-based devices remains an ongoing challenge. Many researchers have addressed this by developing portable versions of conventional optical instrumentation, while others have instead attempted to incorporate part or all the optical detection system on the microfluidic substrate itself. Furthermore, a number of label-free detection techniques were exploited for simplifying the design of optical microfluidic devices. Although fluid handling steps can be further reduced by employing label-free detection, the label-free sensors suffer from limited sensitivity, strict requirements of assay optimization and precise control on differentiation between nonspecific and specific binding especially when testing complex samples.

The future will likely belong to integrated LOC microfluidic devices that possess the desired performance and stability while providing autonomous diagnostics at the point of care, without a need for laboratory analysis. In this regard, chemiluminescence is for instance an optical method offering a good compromise between detection sensitivity, assay time and final device cost. Moreover, as the technology moves close to commercial viability, there will be a need for mass fabrication of the optical microfluidic sensors. While rapid prototyping of PDMS chips is widely exploited in microfluidics, injection molding of thermoplastics is an appropriate fabrication process for mass production. Nevertheless, OPDs may be a cost-effective alternative as integrated optical sensor for LOCs [100,101]. Versatile thin-film (<1 μm) photodetectors employing OPD technology can be fabricated by techniques amenable for mass production.

## Figures and Tables

**Table 1.** Summary of the electrochemical [18], mechanical [19] and optical detection [20] technologies employed in microfluidic devices.

**Method**	**Mechanism**	**Features**
Electrochemical	Measures changes in conductance, resistance, and/or capacitance at the active surface of the electrodes	(+) Real-time detection (∼hundreds seconds range)
		(+) Low-cost microelectrode fabrication
		(+) Widely employed in point-of-care
		(−) Control of ionic concentrations before detection
		(−) Short shelf life

Mechanical	Detection is based on variations of the resonant frequency or surface stress of the mechanical sensor	(+) Monolithic sensor integration
		(+) Label-free detection
		(−) Damping effects in liquid samples
		(−) Detection generally needs around 30 min
		(−) Complex fabrication

Optical	Detects variations in light intensity, refractive index sensitivity, or interference pattern	(+) Minimal sample preparation
		(+) Real-time detection (∼hundreds seconds range)
		(+) Ubiquitous in laboratory
		(−) Conventional opto-instrumentation is expensive
		(−) Set-up complexity

**Table 2.** Up-to-date summary on opto-microfluidic detection methods.

**Optical Detection**	**Sensor Technology**	**Analyte**	**Assay Type**	**Time of Analysis**	**Resolution**	**Point of Care** ^a^	**Ref.**
Fluorescence	CMOS image sensor	*Giardia Lamblia* cysts	Microscopy	∼1 s	Focal plane of 0.8 μm	+	[41]
Fluorescence	CCD camera	Bacterial DNA	PCR	Real time	∼50 CFU/mL ^b^	+	[49]
Fluorescence	Inorganic photodiodes	17-β estradiol	Competitive aptamer assay	∼10 min	0.6 ng/mL	++	[50]
Fluorescence	Organic photodiodes	Alkylphenol polyethoxylates	Competitive immunoassay	∼5 min	2–4 ppb	++	[51]
Chemiluminescence	Microplate reader	Hepatitis B antigen	Capillary immunoassay	25 min	0.3 ng/mL	+	[52]
Chemiluminescence	PMT	Carcinoembryonic antigen	Sandwich immunoassay	-	20 pg/mL	+	[53]
Chemiluminescence	CCD camera	Staphylococcal enterotoxin B	Sandwich immunoassay	>60 min	0.1 ng/mL	+	[54]
Chemiluminescence	Inorganic photodiodes	Anti-HRP antibody	HRP-luminol reactions	>60 min	0.2 amol	++	[55]
Chemiluminescence	Inorganic photoconductor	Streptavidin	HRP-luminol reactions	Real time	4.76 nM	++	[56]
Chemiluminescence	Organic photodiodes	Staphylococcal enterotoxin B	Sandwich immunoassay	60–70 s	0.5 ng/mL	+++	[57]
Absorbance	CMOS image sensor	Glucose	Colorimetric enzyme assay	Real time	-	++	[58]
Absorbance	CCD camera	Cancer HE4 biomarker	Colorimetric sandwich ELISA	5 h	19.5 ng/mL	+	[59]
Absorbance	Visual/no sensor	*E. coli*; *Salmonella*, *Listeria*	Colorimetric enzyme assay	12 h	10 CFU/cm^2^	+	[60]
SPR	Infrared camera	Bovine serum albumin	Protein adsorption	Real time	∼12 pg/mm^2^	+	[61]
SPR	CCD camera	human α-thrombin	Label-free immunoassay	Real time	∼5 nM	++	[62]
SPR	CCD camera	Bacterial rRNA	Hybridization of target RNA	3 h	0.45 fM	+	[63]


a Potential point-of-care uses: +++ High; ++ Moderate; + Low;

b CFU-Colony Forming Unit.
